# MicroRNAs as regulators of cell death mechanisms in amyotrophic lateral sclerosis

**DOI:** 10.1111/jcmm.13976

**Published:** 2019-01-04

**Authors:** Delia Gagliardi, Giacomo P. Comi, Nereo Bresolin, Stefania Corti

**Affiliations:** ^1^ Dino Ferrari Centre Neuroscience Section Department of Pathophysiology and Transplantation (DEPT) Neurology Unit IRCCS Foundation Ca’ Granda Ospedale Maggiore Policlinico University of Milan Milan Italy

**Keywords:** amyotrophic lateral sclerosis, apoptosis, microRNAs, motor neurons, necroptosis, therapy

## Abstract

Amyotrophic lateral sclerosis (ALS) is a progressive neurodegenerative disorder affecting upper and lower motor neurons (MNs), resulting in paralysis and precocious death from respiratory failure. Although the causes of ALS are incompletely understood, the role of alterations in RNA metabolism seems central. MicroRNAs (miRNAs) are noncoding RNAs implicated in the regulation of gene expression of many relevant physiological processes, including cell death. The recent model of programmed cell death (PCD) encompasses different mechanisms, from apoptosis to regulated necrosis (RN), in particular necroptosis. Both apoptosis and necroptosis play a significant role in the progressive death of MNs in ALS. In this review, we present key research related to miRNAs that modulate apoptosis and RN pathways in ALS. We also discuss whether these miRNAs represent potential targets for therapeutic development in patients.

## 1 INTRODUCTION

Amyotrophic lateral sclerosis (ALS) is a fatal neurological disease characterized by the progressive degeneration of both upper and lower motor neurons (MNs), which results in muscle paralysis and ultimately death from respiratory failure within a median of 3 years.^1^ At present, no effective therapy is available for ALS. Riluzole and Edaravone are the sole two drugs approved for ALS but only modestly slow disease progression.^1^, ^2^

Patients affected by idiopathic ALS and without a family history are classified as sporadic (sALS). A fraction of cases (approximately 10%) are familial (fALS), because of mutations in genes involved in a wide range of cellular functions, encompassing roles in motor neuronal and non‐neuronal cells.^3^, ^4^

Since the first identification, in 1993, of superoxide dismutase 1 (SOD1) gene mutations as being responsible for some forms of fALS,^5^ many other genes linked to ALS have been identified.^4^ The most frequent altered gene in both sALS and fALS is *C9ORF72*.^6^ Other relevant genes are *TAR DNA‐binding protein (TARDBP)*, encoding for TDP‐43, and *fused in sarcoma (FUS)*, which encode for proteins implicated in the regulation of RNA processing and expression.^4^ Intracellular inclusions of TDP‐43 are the main pathological substrate of sporadic and familial forms of ALS, except for those associated with *SOD1* mutations.^7^, ^8^, ^9^

Understanding the etiology of ALS and the factors that influence its progression is crucial for the implementation of effective therapeutic strategies that are urgently needed. Although specific causes leading to ALS are unknown, different cellular mechanisms were proposed to mediate MN degeneration, such as glutamate excitotoxicity, mitochondrial dysfunction, protein aggregation, proteasomal and autophagic dysfunction, neuroinflammation, altered axonal transport, and impaired RNA metabolism.^1^ In this context, the role of alterations of RNA metabolism seems particularly central, especially considering that *TDP43* and *FUS* are key components of coding and noncoding RNA processing.

MicroRNAs (miRNAs) are short noncoding RNAs that exert a pivotal role in the regulation of gene expression of many relevant physiological processes and, also, in MNs.^10^, ^11^ miRNAs bind to Argonaute (AGO) proteins to form a ribonucleoprotein (RNP) complex and recognize complementary sequences of messenger RNA (mRNA) via base‐pairing, inducing the down‐regulation of RNA targets.^10^, ^12^ However, because physiological cellular processes need a complex regulatory mechanism, dysregulation of these molecular pathways, typically occurring in chronic diseases, lead to a similarly complex dysregulation of the miRNA expression profile. Indeed, the alterations of each specific miRNA and, consequently, of pathways in which they are involved are variable in different tissues and different disorders.

The finding that miRNAs are essential to MN physiology and survival, supported by the observation that transgenic mice that do not process miRNAs and show hallmarks of MN degeneration,^13^ prompted the investigation of the role of miRNAs in MN diseases, in particular ALS and type 1 spinal muscular atrophy (SMA) (reviewed by us elsewhere).^14^, ^15^ In both these conditions, the miRNA expression profile was shown to be deregulated both in the central nervous system (CNS) and peripheral tissues, with ALS displaying a more profound global dysregulation.^14^ Deregulation in the miRNA expression pattern was reported in several tissues from ALS patients, in particular in the spinal cord, brain, blood, and cerebrospinal fluid (CSF), and it was also demonstrated in induced pluripotent stem cells (iPSCs) generated from affected patients.^14^ The human spinal cord miRNA expression profile showed a substantial global down‐regulation in motor neuron disease,^16^ and this alteration was then demonstrated to be particular to MNs.^17^ These findings have been related to the inhibition of DICER, an endoribonuclease involved in the processing of pre‐miRNA molecules.^17^ Remarkably, miRNA reduction was found also in fibroblasts and was confirmed in serum, plasma, CSF, and the motor cortex.^18^, ^19^, ^20^, ^21^, ^22^

The most constant finding in ALS mutant SOD1 rodents is an increase in miR‐9 and miR‐206.^23^, ^24^, ^25^, ^26^ In particular, the miR‐9 level is increased in ALS rodent spinal cords,^24^, ^26^ whereas the miR‐206 level is increased in skeletal muscles in both SOD1 and SMA murine models.^27^ Remarkably, the reduction in miR‐206 has a negative effect on the ALS disease course in mice, suggesting that it could exert a protective role.^23^ Additionally, a constant increase in the miR‐206 level in skeletal muscle^28^, ^29^ and in serum has been described in ALS patients.^25^, ^29^ This finding may be associated with clinical progression, suggesting that miR‐206 may represent a biomarker in motor neuron disorders, particularly ALS.^29^

Furthermore, miRNAs are key elements in the regulation of cell death, which is the crucial event in the pathophysiology of MN disorders. Programmed cell death (PCD), which has been considered an analogue to apoptosis until recently, was actually extended to include other mechanisms of regulated necrosis (RN), specifically necroptosis. Both apoptosis and necroptosis exert a significant role in ALS MN death.^30^, ^31^, ^32^ In fact, although ALS can represent a rather heterogeneous group of diseases, one of the final common pathogenic elements is represented by MN death, which can serve also as a common therapeutic target in addition to the etiology of ALS.

**Figure 1:**
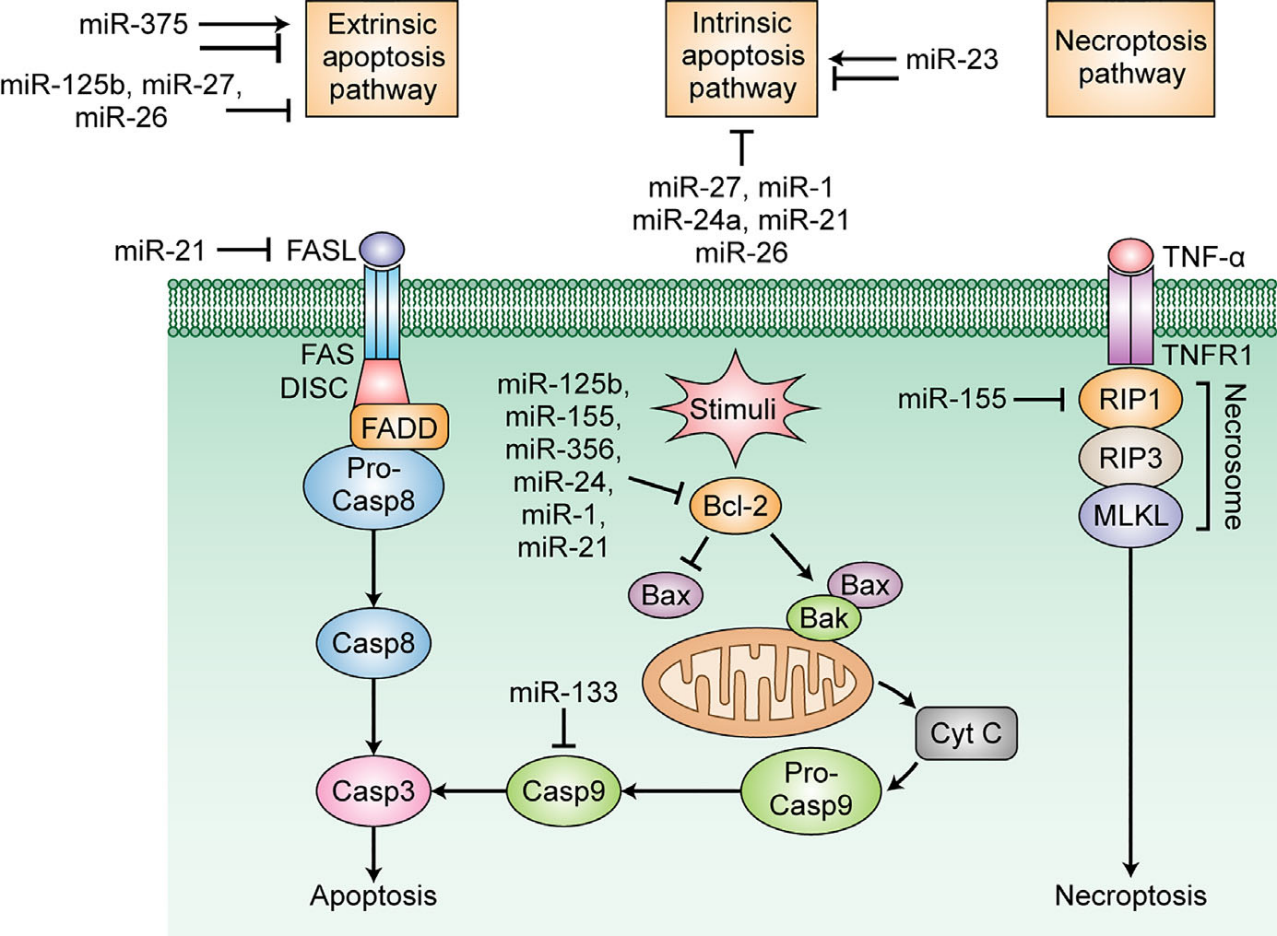
Cell death pathways currently implicated in motor neuron cell death in ALS and related miRNAs. (A) Extrinsic apoptosis. Fas ligand (FASL) activation of the cognate FAS death receptor induces intracellular formation of the death‐inducing signalling complex (DISC), which in turn interacts with Fas‐associated protein with death domain (FADD) through their death effector domains, inducing the recruitment and dissociation of pro‐caspase‐8 from the DISC. Activated caspase‐8 initiates the caspase cascade and leads to apoptosis through caspase‐3 activation. miR‐375 can promote the extrinsic pathway by various mechanisms. miR‐375, miR‐125b, and miR‐27a/b can inhibit the extrinsic pathway by silencing p53. miR‐21 can inhibit the extrinsic pathway by targeting FasL. (B) Intrinsic apoptosis. Following cytotoxic stimuli, pro‐apoptotic BH3‐only proteins silence the anti‐apoptotic protein BCL2, effectively allowing BAX and BAK to dimerize. This dimerization makes the outer mitochondrial membrane permeable and allows cytochrome_c (cyt_c) release into the cytoplasm. The cyt_c binding to apoptotic protease activating factor‐1 (Apaf‐1) prompts formation of the apoptosome, which activates caspase‐9 and the caspase cascade, in which apoptosis is ultimately driven by caspase‐3 activation. MiR‐125b, miR‐155, miR‐365, miR‐24, miR‐1, and miR‐21 promote apoptosis by silencing BCL2. MiR‐133a inhibits apoptosis by silencing caspase‐9. MiR‐23, miR‐27, miR‐1, miR‐21, and miR‐26 inhibit intrinsic apoptosis, and miR‐23 has also pro‐apoptotic properties. (C) Necroptosis. Necroptosis is favoured by low levels of caspase‐8, combined with sufficient concentrations of receptor‐interacting protein 3 (RIP3) and mixed lineage kinase domain like pseudokinase (MLKL). Following tumour necrosis factor receptor 1 (TNFR1) stimulation by tumour necrosis factor (TNF), RIP1 interacts with RIP3, and its phosphorylation is activated MLKL. They form a complex called the necrosome, which causes cell membrane rupture. miR‐155 inhibits necroptosis by silencing RIP1

In this review, we present relevant information pertaining to the miRNA role as modulators of apoptosis and RN pathways in ALS (Figure 1, Table 1), considering both their possible pathogenic role and their use as therapeutic targets.

**Table 1:** miRNAs modulating apoptosis and necroptosis pathways in ALS

miRNA	Target molecule	Regulatory effect	Apoptosis/Necroptosis pathways	Data in ALS
miR‐125b	BCL2	Pro‐apoptotic^35^	Intrinsic apoptosis	Up‐regulated in the CNS of SOD1G93A mice^38^, ^39^
	7sl lncRNA	Pro‐apoptotic^37^		
		Anti‐apoptotic^36^		
	p53		Extrinsic apoptosis	
miR‐155	BCL2	Pro‐apoptotic^35^	Intrinsic apoptosis	Up‐regulated in microglia of SOD1G93A mice^39^ Increased in the ALS spinal cord: fivefold in mice and twofold in humans^40^ Anti‐miR‐155 prolonged survival in SOD1 mice^40^, ^41^
		Anti‐necroptotic^77^		
	RIP1		Necroptosis	
miR‐365 and miR‐24	BCL2	Pro‐apoptotic^43^	Intrinsic aptoptosis	Dysregulated in microglia ALS mice^39^
miR‐23a/b	Apaf‐1	Anti‐apoptotic^44^	Intrinsic aptoptosis	Increased in skeletal muscle of ALS patients; influence on mitochondrial function^28^
		Pro‐apoptotic^45^		
	XIAP		Intrinsic aptoptosis	
miR‐27a/b	Apaf‐1	Anti‐apoptotic^44^	Intrinsic apoptosis	Reduced in serum of ALS patients^46^
		Pro‐apoptotic^68^		
	FADD		Extrinsic apoptosis	
miR‐133a	Caspase‐9	Anti‐apoptotic^47^	Intrinsic aptoptosis	Reduced in in SOD1G93A mice muscle^26^
miR‐1	Unknown	Anti‐apoptotic^47^	Intrinsic aptoptosis	Reduced in in SOD1G93A mice muscle^26^
	BCL2, HSP60, HSP70	Pro‐apoptotic^48^	Intrinsic aptoptosis	
miR‐29a	MCL‐1, P85a, CDC42	Pro‐apoptotic^48^, ^50^	Intrinsic aptoptosis	Reduced in SOD1G93A mice muscle^26^ Increased in the lumbar spinal cord of ALS SOD1G3A mice^49^
miR‐375	cIAP? cFLIP‐L? p53, ELAVL4	Pro‐apoptotic^69^	Extrinsic apoptosis	Reduced in ALS iPSCs‐derived MNs carrying *FUS* mutations^71^
		Anti‐apoptotic^70^		
miR‐34a	BCL2	Pro‐apoptotic^56^, ^57^	Intrinsic apoptosis	Decreased in SOD1G93A mice brainstem and spinal cord^62^
	SIRT1			
miR‐26	MTDH EZH2	Pro‐apoptotic^59^	Intrinsic and extrinsic apoptosis	Decreased in SOD1G93A mice brainstem and spinal cord^62^
miR‐21	BCL2, TGFBR2, Pdcd4	Anti‐apoptotic^65^	Intrinsic apoptosis	Increased in spinal cord and decreased in brainstem of SOD1G93A mice^62^
	PPARα, FASL		Extrinsic apoptosis	
	PTEN		Intrinisc and extrinsic apoptosis	

ALS: Amyotrophic lateral sclerosis; Apaf‐1: apoptotic protease activating factor‐1; CDC42: cell division control protein 42 homolog; cFLIP‐L: cellular FLICE (FADD‐like IL‐1β‐converting enzyme)‐inhibitory protein; cIAP: Cellular inhibitor of apoptosis 1; CNS: central nervous system; FADD: Fas‐associated protein with death domain; FASL: Fas Ligand; ELAVL4: ELAV‐like protein 4; EZH2: enhancer of zeste homolog 2; iPSCs: induced pluripotent stem cells; lncRNA: long noncoding RNA; miRNA: micro‐RNA; MCL‐1: induced myeloid leukaemia cell differentiation; MNs: motor neurons; MTDH: Metadherin; Pdcd4: programmed cell death 4; PPARα: peroxisome proliferator activated receptor alpha; PTEN: phosphatase and tensin homolog; RIP1: receptor‐interacting protein 1; SIRT1: silent information regulator 1; TGFBR2: transforming growth factor beta receptor II; XIAP: X‐linked inhibitor of apoptosis protein.

## 2 miRNAs AND INTRINSIC APOPTOSIS

Apoptosis is classically defined as PCD, or intracellular processes‐mediated cell death.

The term ‘intrinsic apoptosis’ refers to mitochondrial‐related apoptosis. It can be triggered by intracellular pathogenic injuries, such as oxidative stress or DNA damage, and it is regulated by the BCL2 protein.^32^, ^33^, ^34^ Under normal conditions, the anti‐apoptotic protein BCL2 inhibits apoptosis by blocking dimerization of the pro‐apoptotic molecules BAX and BAK.^33^, ^34^ In the presence of pathological stimuli, the BCL2 protein is hindered by the pro‐apoptotic BH3‐only proteins, favouring BAX and BAK dimerization on the outer mitochondrial membrane. BAX and BAK binding promotes permeabilization of the mitochondrial outer membrane and cytochrome c (cyt c) release into the cytoplasm. Cytosolic cyt c binds apoptotic protease activating factor‐1 (Apaf‐1) to facilitate formation of the multiprotein complex the apoptosome, which stimulates caspase‐9. Caspase‐9 activation further facilitates the caspase cascade, including caspase‐3, and the completion of apoptosis.^33^, ^34^

Intrinsic apoptosis can be controlled by several miRNAs, and a group of miRNAs are also dysregulated in ALS. Among them, miR‐125b and miR‐155 were found to display both pro‐apoptotic and anti‐apoptotic properties. Specifically, they participate in BCL2 inhibition in response to CD154 (CD40 ligand) in human leukemic B‐cells,^35^ thus activating the apoptotic process.

Nevertheless, miR‐125b is known as a negative regulator of p53,^36^ which in turn is a mediator of apoptotic pathways during cell stress. Finally, miR‐125b was reported to target the 7sl long noncoding RNA (lncRNA) as well,^37^ and because lncRNA is a p53 repressor, this may also contribute to its pro‐apoptotic properties.

miR‐125b was demonstrated to be up‐regulated in the CNS of SOD1‐G93A mice, correlating with neurodegeneration and astrocytosis.^38^ miR‐125b and miR‐155 levels were also augmented in the microglia of SOD1G93A mice, where they promote neuroinflammation.^39^ Increased miR‐155 expression was demonstrated in the ALS spinal cord, with a fivefold increase in rodent and twofold increase in patients samples.^40^ Anti‐miR‐155 prompted generalized de‐repression of mRNA targets in peritoneal macrophages and was found to disseminate in the mouse brain and spinal cord after intraventricular delivery. Treatment of SOD1‐G93A mice with anti‐miR‐155 significantly increased disease duration by 15 days and life length by 10 days.^40^ These data were confirmed by another set of experiments demonstrating that intraventricular delivery of anti‐miR‐155 reversed the expression of microglial miR‐155 targeted genes and that peripheral anti‐miR‐155 administration extended the lifespan.^41^ Moreover, abnormal microglia and monocyte signatures were recovered after genetic deletion of microglial miR‐155, and the lifespan of SOD1 mice increased by 51 days in females and 27 days in male animals.^41^ miR‐155 appears to be consistently over‐expressed in ALS in different studies and may be a biomarker for early disease identification.^42^

Several other miRNAs, including miR‐195, miR‐24, and miR‐365‐2, regulate the anti‐apoptotic protein BCL2 by targeting the 3′‐UTR.^43^ Up‐regulation of these molecules promotes PCD in breast cancer cells otherwise resistant to apoptosis. Additionally, miR‐365 and miR‐24 are dysregulated in microglia ALS.^39^

Apaf‐1, a key element of the apoptosome, is regulated by four RNA molecules (miR‐23a/b and miR‐27a/b), which assemble, forming two miRNA clusters, miR‐23a‐27a‐24 and miR‐23b‐27b‐24.^44^ MiR‐23a/b and miR‐27a/b inhibited Apaf‐1 expression in vitro, and their levels were inversely correlated with Apaf‐1 levels in the mouse cortex. Indeed, whereas hypoxic injuries induce the down‐regulation of miR‐23b and miR‐27b and increase Apaf‐1 expression in mouse neurons, overexpression of the two miRNAs in transgenic mice suppressed the apoptosis of neural cells induced by hypoxia.^44^ These findings indicate that these miRNAs may represent potential therapeutic strategies in neuronal apoptosis‐related diseases.

By contrast, it was shown that miR‐23a can exert a pro‐apoptotic role by inhibiting X‐linked inhibitor of apoptosis protein (XIAP), which binds and inhibits caspase 3, 7, and 9. Specifically, various miRNAs, such as miR‐23a, miR‐24, and the miR‐130 cluster, bind to the XIAP 3′‐UTR, inducing apoptosis.^45^

Compared to healthy controls, miR‐23a, along with miR‐29b, miR‐206, and miR‐455, was up‐regulated in the skeletal muscle of ALS subjects.^28^ In this context, miR‐23a caused a decrease in peroxisome proliferator‐activated receptor γ coactivator‐1α (PGC‐1α) protein expression in ALS tissue. This potentially has a negative effect on skeletal muscle mitochondrial function.^28^ miR‐27a was found diminished in the serum of ALS patients compared to healthy individuals.^46^

miR‐133 and miR‐1 are normally present in adult skeletal muscle and cardiomyocytes. miR‐133 binds caspase‐9 at its 3′‐UTR binding site, and it's up‐regulation by ischaemic postconditioning or through miRNA mimics prevents apoptosis induced by ischaemia‐reperfusion in rat hearts.^47^ miR‐1 is proposed to promote intrinsic apoptosis by regulating BCL2, HSP60 and HSP70^48^; however, it is reported that along with miR‐133a, it plays a key role in protecting myocardial cells in case of damage, suppressing apoptosis‐associated genes.^47^

Several physiological or pathological events impact miRNA expression, in turn activating or inhibiting the intrinsic apoptotic pathway. For example, in ischaemia‐reperfusion heart damage, altered levels of several miRNAs, such as miR‐1, miR‐21, miR‐29, and miR‐133, modify the expression of several miRNA‐targeted genes, including *Bcl‐2, PTEN, Mcl‐1, HSP20, HSP60, HSP70, LRRFIP1, Pdcd4, and Sirt‐1*, which can independently or jointly prompt intrinsic apoptosis and influence tissue damage.^48^

Several of the above cited miRNAs were found altered in SOD1G93A rodent muscle tissue; in particular, miR‐1, miR‐29, and miR‐133 were reduced compared to wild‐type. This local expression can influence the CNS mRNA patterns, particularly those linked with the myelination events in the spinal cord.^26^ Otherwise, higher expression of miR‐29a, which is specific for the CNS, has been observed from postnatal day 70 in the lumbar tract of the SOD1G3A mouse spinal cord compared to controls.^49^ After miR‐29a knockdown obtained by a one‐time intraventricular administration of a miR‐29a‐specific antagomir in the CNS, miR‐29 down‐regulation did not show amelioration in terms of disease progression and motor performance, despite a slight increase in lifespan in male mice.^49^ Indeed, up‐regulation of miR‐29 caused a reduction in the induced myeloid leukaemia cell differentiation (MCL‐1) protein, which, when expressed, has an anti‐apoptotic role similar to BCL2.^50^

The miR‐34 family comprises three miRNAs encoded by two different loci: the miR‐34a locus is located on chromosome 1, whereas the miR‐34b/34c cluster is located on chromosome 11. Although the latter is expressed in lungs, miR‐34 is ubiquitous, with the greatest level in the brain.^51^ mir‐34a has a role in cell death, particularly in caspase‐dependent apoptosis, which has been extensively recognized.^52^ At first, ectopic expression of miR‐34a in neuroblastoma cell lines (in which its levels are normally decreased) was found to induce apoptosis.^53^ Then, Chang and colleagues reported that p53 induced the up‐regulation of miR‐34a, which in turn regulates genes related to cell proliferation, DNA repair and apoptosis, thus playing a crucial role in p53‐dependent and independent apoptosis.^54^ These data were confirmed by Bommer et al^55^, who demonstrated that miR‐34a is one of the effectors of the p53 tumour suppressor function and that BCL2 is one of its target genes. Moreover, BCL2 was found to be down‐regulated by miR‐34a in a mouse model of Alzheimer's disease, and miR‐34a repression in cell lines resulted in up‐regulation of the BCL2 protein and a decrease in caspase 3 levels.^56^ Silent information regulator 1 (SIRT1) is a deacetylase that regulates apoptosis induced by oxidative stress and DNA damage by inactivating several molecular targets, including p53. miR‐34a was shown to inhibit SIRT1, thus promoting activation of acetylated p53 and leading to increased expression of its transcriptional target p21 and PUMA.^57^ Therefore, miR‐34a and p53 may promote apoptosis by regulating each other through a positive feedback loop.

Similar to miR‐34, miR‐26 is an apoptosis‐involved miRNA that exhibits different expression profiles in different biological and pathologic processes, such as growth, development, and tumourigenesis.^58^ It was found down‐regulated in several types of tumours, where it likely exerts a tumour‐suppressor function, and it is over‐expressed in gliomas, where it promotes cell growth and proliferation by regulating one of its target, *PTEN*. In particular, miR‐26 prompted activation of caspase‐9 and caspase‐8, inducing both intrinsic and extrinsic apoptosis, respectively, in human breast cancer cells by targeting *EZH2* and *MTDH*.^59^ The latter gene, which encodes for a histone‐methyltransferase enzyme involved in transcriptional repression, is also silenced during myogenesis, suggesting a role for miR‐26 in the regulation of proliferation and differentiation.^60^ Finally, through autophagy inhibition, miR‐26 increased apoptosis in hepatocellular carcinoma cells.^61^

A decrease in miR‐34a and miR‐26b levels was found in the spinal cord and brainstem nuclei of SOD1‐G93A rodent models.^62^

Finally, given its constant over‐expression in numerous cancers, miR‐21 has been widely studied in oncology for its anti‐apoptotic properties. At first, miR‐21 knockdown was shown to increase apoptotic cell death in murine models and in human glioblastoma cells.^63^, ^64^ Then, a number of tumour‐suppressor genes implicated in apoptosis were identified as targets of miR‐21 regulation. Among them are molecules involved in intrinsic apoptosis, such as the anti‐apoptotic protein BCL2, transforming growth factor beta receptor II (TGFBR2), which participates in the pro‐apoptotic signalling of TGF‐beta, programmed cell death 4 (Pdcd4), a pro‐apoptotic gene found up‐regulated in hepatocellular cancer, and other proteins participating in the extrinsic pathway, such Fas‐ligand (FASL) and peroxisome proliferator activated receptor alpha (PPARα).^65^ Phosphatase and tensin homolog (PTEN), one of the most important miR‐21 targets, is a mediator of both intrinsic and extrinsic apoptosis because it has a role in both mitochondrial and tumour necrosis factor (TNF) signalling.

Although its role in ALS has not been completely recognized, over‐expression in the spinal cord and down‐regulation in the brainstem were described in mouse models.^62^ Indeed, it may play a function in glial interplay and the inflammatory response because it regulates astrocyte hypertrophy and scar formation after a spinal cord injury.^66^

## 3 miRNA AND EXTRINSIC APOPTOSIS

Extrinsic apoptosis is triggered by the binding of extracellular ligands to specific surface receptors, called death receptors (DRs). The most well‐characterized DRs belong to the TNF family, such as TNF receptor 1 (TNFR1) and Fas. Ligand binding and activation cause the assembly of the death‐inducing signalling complex (DISC), which in turn cooperates with the adaptor protein Fas‐associated protein with death domain (FADD), mediated by their death effector domains (DEDs). This interplay leads to the release and cleavage of pro‐caspase‐8 that once activated, prompts a caspase signalling cascade that ultimately elicits apoptosis. Several miRNAs can selectively recognize and inhibit death ligands and receptors.

As previously noted, miR‐125b targets *p53*, preventing cellular death, likely through a nonintrinsic‐cell mechanism.^67^

In human embryonic kidney cells, the miRNA cluster miR‐23a‐27a‐24 may separately promote either caspase‐dependent or independent apoptosis by silencing FADD expression.^68^ In particular, FADD is expected to be the target of miR‐27a based on a bioinformatics target prediction algorithm; its levels are increased proportionally to a low level of miR‐27a, and its expression was found to be directly inhibited by miR‐27a binding to its target sequence.^68^ Moreover, the miR‐23a‐27a‐24 cluster enhances TNFα cytotoxicity by increasing the expression of TNF receptor associated factor 2 (TRAF‐2) protein, which is an effector of TNFR1 signalling. As discussed above, serum miR‐27a was reduced in ALS patients.^46^

miR‐375 also increases TNFα‐induced apoptosis, although the mechanisms are unclear, although reductions in both cellular inhibitor of apoptosis 1 (cIAP) and cellular FLICE (FADD‐like IL‐1β‐converting enzyme)‐inhibitory protein (cFLIP‐L) by miR‐375 have been described.^69^

miR‐375 is implicated in the motor neuron disease phenotype, likely playing a protective role.^70^ miR‐375 is implicated in neurogenesis, inducing cell proliferation and protecting MNs from DNA damage‐induced apoptosis.^70^ Moreover, MNs derived from an SMA patient presented low levels of miR‐375, increased expression of p53 protein, and increased susceptibility to apoptosis in response to DNA damage.^70^ Remarkably, it was recently shown that miR‐375 levels were also diminished in ALS induced pluripotent stem cells (iPSCs)‐derived MNs harbouring *FUS* pathogenic mutations.^71^ Accordingly, *FUS* mutant MNs displayed an over‐expression of p53 and other pro‐apoptotic miR‐375 predicted targets, such as ELAV‐like protein 4 (ELAVL4). Indeed, ALS spinal MNs present increased levels of p53 protein, which may be the main driver of neuronal death mediated by apoptotic mechanisms.^72^

Thus, miR‐375 dysregulation is a condition shared by two different motor neuron disorders, SMA^70^ and *FUS*‐ALS^71^ MNs, and miR‐375 was reduced by 40% and 40%‐50%, respectively. The molecular mechanisms of miR‐375 dysregulation in SMA are unknown at present; however, it was hypothesized that *FUS* loss‐of‐function mutations in ALS are responsible for FUS protein discharge from the nucleus and interruption of miR‐375 synthesis.

Moreover, in addition to p53‐mediated apoptosis, miR‐375 reduction may have effects on other relevant pathways. It was associated with an up‐regulation of its target ELAVL4, an RNA‐binding protein implicated in CNS development, functioning, and plasticity.^73^ For this reason, it was proposed that FUS/miR‐375/ELAVL4 signalling could be a regulatory pathway involved in the RNA metabolism of MNs.^71^

## 4 miRNA AND PROGRAMMED NECROSIS

Necrosis was traditionally considered an unprogrammed and premature cell death caused by damage from pathologic external stress, resulting in cell disruption and outflow of cell content from the nucleus and cytoplasm, which in turn can represent pro‐inflammatory signals.^32^ Nevertheless, this hypothesis has been questioned by recent evidence suggesting that necrosis can also be a regulated process, sharing some features with apoptosis. One type of RN is called necroptosis. Similar to apoptosis, necroptosis is a type of PCD and involves DRs of the TNF family. By contrast, it is a caspase‐independent mechanism, induces membrane disruption and the release of cellular contents into the extracellular matrix, and activates the immune response.^32^

This process can be triggered by ligation of TNF‐α to its receptor TNFR1, and it is promoted when caspase‐8 is inhibited or reduced.^74^, ^75^ It is mediated by receptor‐interacting protein 1 (RIP1) and RIP3, which, along with the mixed lineage kinase domain like pseudokinase (MLKL), form a complex called the necrosome that causes the formation of pores in the cell membrane and finally its rupture.^76^

Re et al^30^ suggested that necroptosis participates in MN death in sALS. They demonstrated that in a coculture of MNs derived from human embryonic stem cells with astrocytes obtained from postmortem sALS patient CNS, unlike controls, the sALS astrocytes induced MN toxicity after 7 days. Treatment with a RIP1 antagonist, necrostatin‐1, and *Rip1* shRNA knockdown increased the number of surviving MNs. Furthermore, treatment with necrosulfonamide, an MLKL inhibitor, led to similar improvement. Thus, these results provided evidence that a caspase‐independent form of PCD, necroptosis, has a leading role in MN death in sALS, both in in vitro and in vivo models.^30^ Additional findings in support of necroptosis in motor neuron disorders came from Ito and colleagues, who described the presence of this form of RN, both in cellular and animal models and in postmortem human samples, demonstrating the presence of increased levels of RIP1, RIP3, and MLKL.^31^ miR‐155 recognizes RIP1 as a target and was demonstrated to be over‐expressed after hydrogen‐peroxide administration in cardiomyocyte progenitor cells. Thus, miR‐155 can inhibit necroptosis in a similar manner as necrostatin.^77^ As mentioned above, increased miR‐155 levels were detected in the ALS CNS,^40^ with an unclear effect on necroptosis.

## 5 miR‐9 AND ‐206 AND CELL DEATH

As noted above, the most constant finding in ALS rodent and human tissues is the increase in miR‐9 in the CNS and miR‐206 in skeletal muscle and serum.^23^, ^24^, ^25^, ^26^

MiR‐9 is an miRNA highly expressed in the nervous system, which regulates neurogenesis by inhibiting neural stem cell self‐renewal and promoting neural differentiation and proliferation. As a master regulator of neurogenesis, a potential role for miR‐9 in apoptosis was supported by the observation that miR‐9 down‐regulation significantly increased cell death in the forebrain of *Xenopus* embryos. By contrast, apoptosis did not occur in *Xenopus* embryo hindbrains and in other model systems.^78^ In particular, it was described that an anti‐miR‐9 morpholino causes apoptosis in neural progenitor cells (NPCs) only in the forebrain, with mechanisms that involve the p53 pathway.^79^ Moreover, there is strong evidence for the function of miR‐9 in neurodegeneration, and up‐regulation of this miRNA was reported in postmortem brains of patients affected by Alzheimer's disease and reduced levels in the precocious stages of Huntington's disease. Finally, miR‐9 is down‐regulated in mouse embryonic cell‐derived MNs carrying the SMN1 mutation and modify the expression and dynamics of intermediate filaments.^78^ It is unclear whether the specific effect of miR‐9 up‐regulation in the CNS is positive or detrimental; however, it is uncontroversial that miR‐9 down‐regulation in the spinal cord causes neurofilament dysregulation and MN degeneration.^13^ Additionally, miR‐9 is implicated in cell proliferation and migration. As altered miR‐9 levels were described in different types of cancer, miR‐9 is also likely implicated in tumour formation and development. In consideration of the variety of its targets, miR‐9 may have different effects on several types of cells and tissue, either inducing proliferation or cell death.^78^ Overall, these findings suggest that miR‐9 has different roles in different contexts, and considering its dysregulation in MNs, proper modulation with drugs or gene therapy could represent an interesting challenge.

miR‐206 along with miR‐1 and miR‐133 are among the best characterized human and mouse muscle‐specific miRNAs. They are implicated in various steps of the muscle differentiation processes, including alternative splicing, DNA synthesis, and cell apoptosis.^80^ During development, miR‐1 and miR‐206 inhibit target paired‐box transcription factor 7 (Pax7) and its homolog Pax3, thus restricting satellite cell proliferative potential and promoting their differentiation in myogenic progenitor cells.^81^ Conversely, miR‐1 and miR‐206 down‐regulation provokes Pax7 and Pax3 overexpression and, subsequently, inhibition of myoblast differentiation. In this context, because Pax7 and Pax3 are pro‐survival factors, their down‐regulation by miR‐206 can cause apoptosis. It is well‐known that ALS is associated with higher expression of miR‐206 in muscle tissue, where it plays a protective role, and is necessary for the regeneration of neuromuscular junctions after acute nerve injury.^23^ ALS mouse models knocked‐out for the miR‐206 gene display later and incomplete muscle reinnervation compared to SOD1G93A mice wild‐type for this miRNA.^82^ It was recently reported that compared to controls, ALS patients showed higher miR‐206 levels in plasma, and interestingly, those values were correlated with muscle strength measured by the Medical Research Council (MRC) scale and were predictive of slower progression over 12 months.^29^ In later disease stages, the miR‐206 concentration decreased in muscle and serum, most likely reflecting a loss of muscle fibres. Considering this, miR‐206 could be a biomarker of lower MN involvement and muscle damage in ALS^29^ and an interesting therapeutic target.

However, even if preliminary data in ALS rodents showed a good therapeutic efficacy for its up‐regulation, a proper pharmacological/molecular modulation of miR‐206 may remain difficult in the clinic.

## 6 THERAPEUTIC PERSPECTIVES

In addition to providing interesting and reliable tools for understanding the pathogenic mechanisms underlying ALS and other neurodegenerative disorders, miRNAs represent promising therapeutic intervention strategies because the modulation of a single miRNA molecule interferes with different target genes, thus modifying broad cellular pathways and intervening at several levels in a complex disease such as ALS.

Approaches for miRNA modulation include (a) substitution of down‐regulated miRNAs with synthetic miRNA mimics, which act at the gene‐level inhibiting translation, (b) up‐regulated miRNA blockade and degradation through antisense oligonucleotides (anti‐miRs) and antagomirs, (c) de‐repression of miRNA target genes through miRNA masks, which bind to the 3′UTR of mRNA, hiding the miRNA binding site, and (d) alteration of miRNA expression at the transcription level using small drug inhibitors.^83^, ^84^

Concerns limiting these procedures include the confined biodistribution with systemic delivery and the capacity to pass the blood brain barrier, which can be overcome using intrathecal infusion, and the molecular stability over time. To improve miRNA cellular entry and allow molecular stabilization against degradation, delivery vehicles, such as liposomes, polymeric particles, and viral vectors, have been developed. In this scenario, the adenovirus‐associated virus is one of the most promising carriers.

Because in most cases miRNAs exert their function in multiple pathways, the major issue of miRNA targeted therapy is the risk of influencing the transcription of off‐target tissues, leading to the occurrence of possible side effects.

Thus far, two miRNAs implicated in cell death were tested as possible therapeutic targets in ALS: miR‐155 and miR‐29a. In both cases, as described above, miRNA overexpression was inhibited with an antagomir and a modest amelioration of the phenotype was observed.^40^, ^49^ No major side effects were recorded, although their detection is limited by the reduced lifespan of SOD1G93A mice.

Considering that miR‐375 can preserve MNs from DNA damage‐induced apoptosis via inhibition of p53 and given its down‐regulation in ALS carrying the FUS mutation,^71^ miR‐375 can represent an interesting therapeutic target. In this case, the up‐regulation of miR‐375 should be obtained.

Finally, because a global reduction in miRNA levels is a shared molecular characteristic for different forms of motor neuron diseases, strategies to generally up‐regulate their production, such as enhancing DICER activity through small molecules, such as enoxacin, can be explored.^17^

## 7 CONCLUSION

Cell death, with various mechanisms spanning from apoptosis to necroptosis, appears to be the final manner of MN loss in ALS. In this context, it could represent a key aspect by which therapeutic efforts can be rationally focused, in addition to a perfect knowledge of the primum movens of the disease. As important regulators of cellular processes, including cell death, miRNAs can be potential targets for therapy development for motor neuron disorders. However, because miRNAs can influence several targets within different molecular pathways, their modification with pharmacological agents or gene therapy strategies can be particularly complex. In this apparently difficult scenario, several themes, such as the opportunity to counteract the general depression of miRNAs in ALS or the specific role of several miRNAs (ie, miR‐375, which can inhibit p53‐mediated cell death) can emerge. These aspects, and hopefully new ones, warrant further investigation for the progress of therapeutic strategies for ALS.

## CONFLICTS OF INTEREST

The authors confirm that there are no conflicts of interests.
